# New Rehabilitation Assessment Method of the End-Effector Finger Rehabilitation Robot Based on Multi-Sensor Source

**DOI:** 10.3390/healthcare9101251

**Published:** 2021-09-23

**Authors:** Hongbo Wang, Peng Chen, Yungui Li, Bowen Sun, Ziyu Liao, Baoshan Niu, Jianye Niu

**Affiliations:** 1 Academy for Engineering & Technology, Fudan University, Shanghai 200433, China; wanghongbo@fudan.edu.cn; 2 Parallel Robot and Mechatronic System Laboratory of Hebei Province, Yanshan University, Qinhuangdao 066000, China; cpeng@stumail.ysu.edu.cn (P.C.); lyg@stumail.ysu.edu.cn (Y.L.); 17865199985@163.com (B.S.); 3 Shanghai Clinical Research Center for Aging and Medicine, Shanghai 200040, China; 4 College of Mechanical and Electrical Engineer, Nanjing University of Aeronautics and Astronautics, Nanjing 210016, China; yamamoto@nuaa.edu.cn; 5 State Key Laboratory of Robotics and System, Harbin Institute of Technology, Harbin 150080, China; 20b908052@stu.hit.edu.cn

**Keywords:** End-Effector Finger Rehabilitation Robot, rehabilitation assessment, finger muscle strength, muscle fatigue degree, range of motion, the AHP method

## Abstract

In the process of rehabilitation, the objectivity and the accuracy of rehabilitation assessment have an obvious impact on the follow-up training. To improve this problem, using a multi-sensor source, this paper attempts to establish a comprehensive assessment method of the finger rehabilitation effect, including three indicators of finger muscle strength, muscle fatigue degree, and range of motion. Firstly, on the basis of the fingertip pressure sensor of the End-Effector Finger Rehabilitation Robot, a mathematical model of finger muscle strength estimation was established, and the estimated muscle strength was scored using the entropy weight method. Secondly, using an sEMG signal sensor, a fatigue monitoring system was designed in the training process, and the fatigue degree was determined on the basis of the change trend of the eigenvalues of MAV and RMS. Lastly, a human–machine motion coupling model was established, and the joint range of motion acquisition and scoring model were obtained on the basis of the motor encoder. According to the above three indicators, using the AHP assessment method to establish a comprehensive rehabilitation assessment method, the effectiveness of the method was verified by experiments. This paper provides a potential new idea and method for objective, accurate, and convenient assessment of finger function rehabilitation, which is of positive significance for alleviating the burden on rehabilitation doctors and improving rehabilitation efficiency.

## 1. Introduction

Rehabilitation assessment is conducted to determine the nature, location, severity, development trend, scope, and prognosis of dysfunction in patients with disabilities [1,2]. It can reflect the situation of dysfunction in patients and lay a scientific foundation for the formulation and implementation of a rehabilitation treatment plan [3,4]. Therefore, it is necessary to conduct an objective, accurate, and convenient assessment in the process of rehabilitation training.

In the field of rehabilitation medicine, the focus of rehabilitation assessment is the assessment of limb motion function [5,6]. At present, the assessment method is usually evaluated by therapists using a clinical assessment scale, including the are Brunnstrom assessment method, Fugl-Meyer assessment (FMA) scale, and Barthel index [7,8]. The FMA method is recognized as one of the most widely used rehabilitation assessment methods. The FMA method has the advantages of detailed content, as well as high assessment reliability and sensitivity [9], but a single assessment of patients takes a long time, and it is mainly subjectively evaluated by rehabilitation doctors, which cannot ensure objective and unified results [10].

Robot-derived measurement technology adds a new dimension to the assessment of motion function [11]. Through the sensor hardware and control system software on the rehabilitation robot, quantitative analysis can be realized, rehabilitation efficiency can be improved, cost can be reduced, and an accurate, objective, and real-time rehabilitation assessment method has become a possibility and a trend [12,13].

In recent years, it has become an active topic to seek more excellent rehabilitation assessment methods based on rehabilitation robots [14,15]. Some assessment methods have been developed. Wu [16] designed two tasks of “following the circle” and “crossing the tunnel” to evaluate and classify healthy people and stroke patients in Brunnstrom phase VI using a neural network, but this method is more suitable for patients at the later stage of rehabilitation. Kurillo [17] predicted the three-dimensional (3D) reachable spatial surface area using Kinect-captured data to evaluate the upper-limb function of patients with facial shoulder brachial muscular dystrophy. Bai [18] collected the upper-limb movement information through Kinect, calculated the upper-limb reachable space, and used the adaptive network fuzzy inference system to evaluate the patient’s upper-limb rehabilitation training results; however, once the camera of the motion capture system in the above two methods is calibrated successfully, it cannot be moved casually; thus, they are not portable and efficient. Gao [19] studied a three-degree-of-freedom upper-limb exoskeleton real-time rehabilitation training system based on a surface electromyography (sEMG) signal. After collecting the sEMG signal, four elbow movements can be identified and evaluated by decision tree algorithm. Compared with the traditional rehabilitation assessment methods, this method has more specific assessment results, but relying only on the sEMG signal which is easil disturbed can lead to poor stability. Su [20] combined sEMG and inertia information with clinical rehabilitation assessment indices to realize a comprehensive and quantitative upper-limb rehabilitation state assessment and database system. However, the assessment system has a small number of test patients and needs to be further improved for clinical practice. Antonella [21] proposed a multi-parameter method to evaluate the rehabilitation of patients, including sEMG, electroencephalogram (EEG), kinematics, and clinical scale. This multisource method can better characterize the rehabilitation of patients, but it is highly complex and requires the assistance of professionals. All these measurement methods have their own advantages; however, at present, most robot rehabilitation assessment technologies still need the assistance of professionals, and there is a lack of large samples and high-quality long-term clinical studies proving their accuracy and reliability [22,23].

Generally speaking, there are no perfect and standardized assessment methods, and this also applies to the rehabilitation assessment of finger function [24]. Using the innovative design of the End-Effector Finger Rehabilitation Robot (EFRR), combined with multisource information such as finger contact pressure, sEMG signal, and finger joint range of motion, this paper formulates a comprehensive rehabilitation assessment method suitable for this system, which has the characteristics of miniaturization and home use without a physician’s assistance. It provides a new idea and possibility for the objective and real-time automatic rehabilitation assessment of finger motion function.

## 2. Materials and Methods

### 2.1. Mechanism Design of EFRR

**Figure 1:**
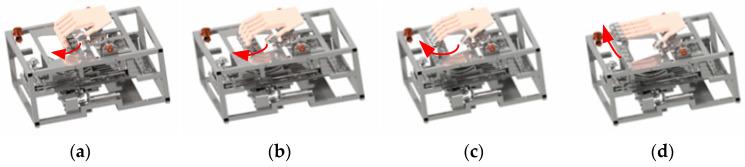
Flexion/extension training: (**a**) start position; (**b**) middle position 1; (**c**) middle position 2; (**d**) end position.

To meet the rehabilitation needs of patients with finger dysfunction, a finger rehabilitation robot was designed on the basis of the physiological structure and movement characteristics of fingers. The rehabilitation robot mainly includes four parts: four-finger flexion/extension assembly, four-finger adduction/abduction assembly, thumb movement assembly, and frame. It can realize the flexion/extension and adduction/abduction training of left or right fingers [25]; the action decompositions of the training process are shown in Figure 1.

**Figure 2:**
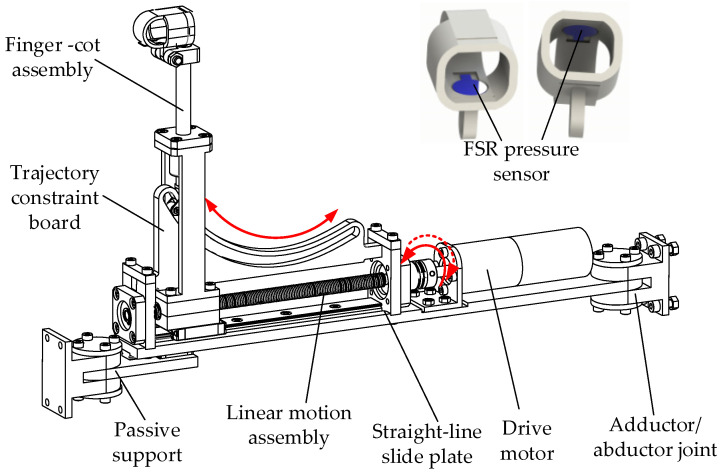
Flexion/extension motion component.

As shown in Figure 2, the four-finger flexion/extension motion component comprises four identical single-finger flexion/extension motion components with modular thinking. Considering the natural trajectory of finger flexion/extension, the circular arc trajectory constraint board was designed to realize the finger movement constraint. The finger-cot assembly is driven to move back and forth through a linear motion module composed of a lead screw and a linear slider. Force-sensing resistor (FSR) pressure sensors are also installed in the finger-cot to detect fingertip pressure during movement. In addition, the motion track constraint module can change according to the human percentile to meet the needs of people with different finger sizes.

### 2.2. Function Composition of EFRR

**Figure 3:**
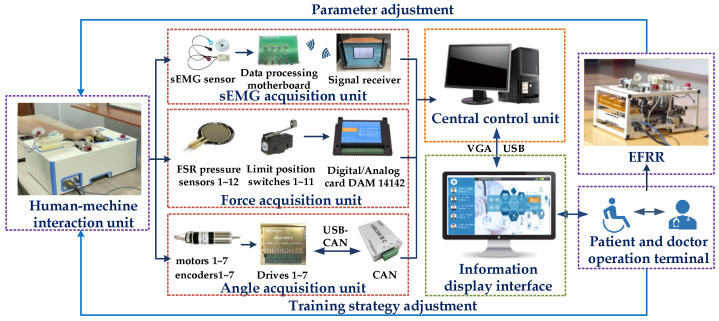
Functional block diagram of EFRR.

According to the function of EFRR, the electrical control system of the robot mainly includes the central control unit, human–machine interaction unit, patient and doctor operation terminal, and data acquisition unit, as shown in Figure 3.

During the rehabilitation training of the human–machine interaction unit, the data acquisition units collect the information from the sEMG, force sensors, and motor encoders, and the central control unit processes the information. The display interface reflects the rehabilitation evaluation on the touch screen. Patients and rehabilitation doctors can adjust the parameters to set the corresponding training strategy so as to realize the closed-loop rehabilitation training of EFRR.

## 3. Results

### 3.1. Muscle Strength Assessment Based on Fingertip Pressure

#### 3.1.1. Pressure Detection of the Fingertip

The rehabilitation effect of patients is an important index to evaluate the treatment plan [26]. Therefore, this paper proposes a rehabilitation assessment method based on fingertip pressure. A finger strength estimation model based on fingertip pressure was established. Using this model, the muscle strength of people can be estimated.

**Figure 4:**
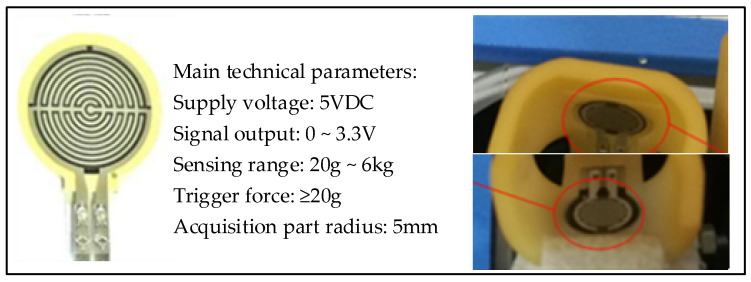
FSR pressure sensor.

After rehabilitation treatment, the rehabilitation effect of patients is significant for the formulation and adjustment of the follow-up rehabilitation treatment plan [27]. Using this model, people’s muscle strength can be estimated, and the estimated muscle strength can be scored by the entropy weight method. Finally, the finger can be graded according to the score value to realize the assessment of the rehabilitation effect [28]. An FSR thin-film pressure sensor was used to collect fingertip pressure, and its shape and detailed parameters are shown in Figure 4.

**Figure 5:**
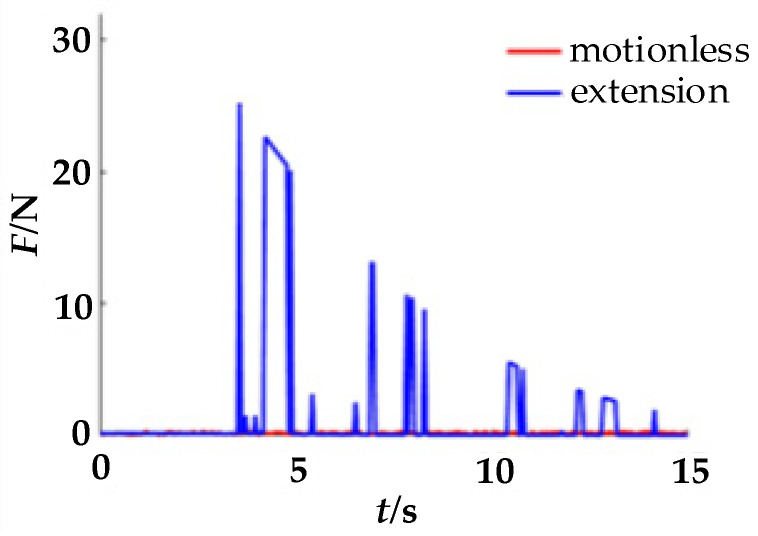
Upper-side piezoelectric signal.

**Figure 6:**
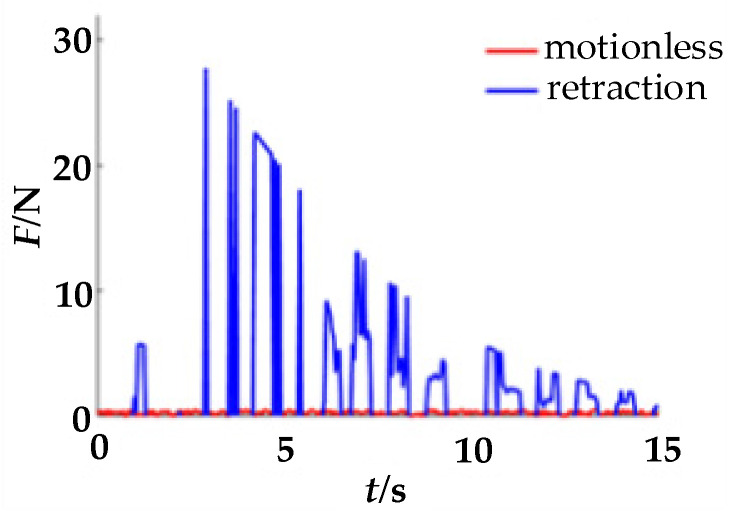
Lower-side piezoelectric signal.

During the experiment, the acquisition equipment needs to first be calibrated. When the finger is in a natural state in the finger cot, the zero coordinates of the sensors on the upper and lower sides will be inconsistent due to the influence of gravity. The pressure sensor on the lower side will have a smaller force signal; hence, it needs to first be compensated for in a positive direction. At the same time, since the output of the piezoelectric signal is a voltage signal, it also needs to be converted into a force signal. Therefore, calibration is required before collecting signals. When a single finger is at rest and there is an extension movement, the pressure information on the upper side of the finger-cot is as shown in Figure 5. When a single finger is at rest and there is a flexion movement, the pressure information on the lower side of the finger-cot is as shown in Figure 6.

As can be seen from Figure 5 and Figure 6, with the extension and flexion movements, the pressure signals converted from the piezoelectric signals on the upper and lower sides gradually decrease with time.

#### 3.1.2. The Calculation of Finger Muscle Strength

**Figure 7:**
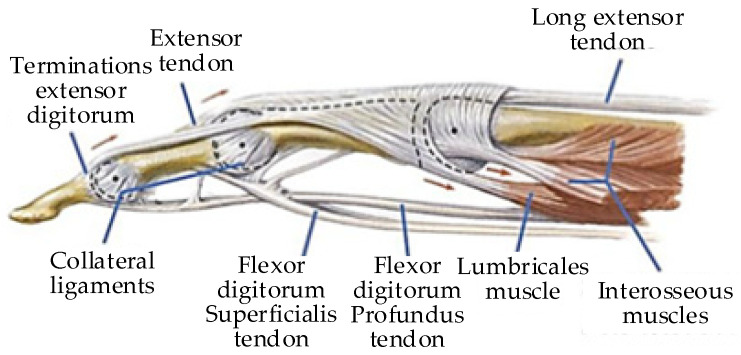
Finger anatomy [32].

The anatomical structure of the finger is shown in Figure 7. The main muscle groups are the flexor digitorum profundus tendon (FDP), flexor digitorum superficialis tendon (FDS), long extensor tendon (LE), and interosseous muscles [29]. Therefore, the muscle strength of the finger is actually the muscle strength of the driving muscle groups. This calculation estimates the muscle strength, and there is a certain error [30]. The existing calculation methods generally estimate the finger muscle strength by measuring other parameters [31].

**Figure 8:**
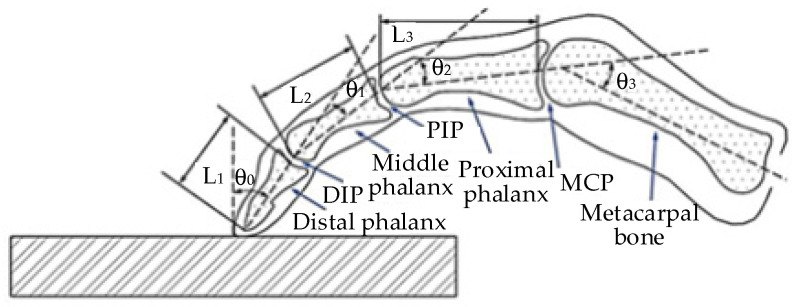
Finger joints and angles.

**Figure 9:**
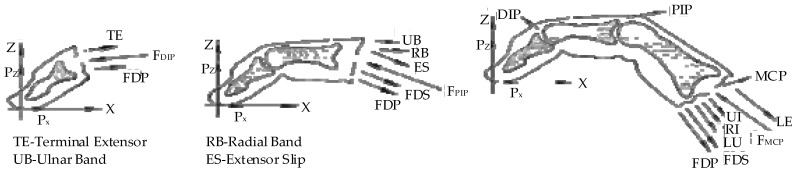
Finger biomechanical relationship.

In this paper, a mathematical model of finger muscle strength was established by measuring the contact force of the fingertip. When the fingers are conducting flexion/extension exercises, they can be set as a plane motion [33]. The joints (distal phalanx (DIP), proximal phalanx (PIP), and metacarpophalangeal phalanx (MCP)) and angles of a finger are shown in Figure 8 [34]. The biomechanical relationship of each finger joint is shown in Figure 9 [35]. The finger muscle strength estimated in this paper is the muscle strength of six muscle groups: LE, ulnar interosseous (UI), radial interosseous (RI), lumbricales muscle (LU), FDS, and FDP. The balanced equation of fingers can be established, which is the force/torque balance equation of three joints.

(1) $$MCP\left\{ \begin{array}{l} F_{FDP}\sin\left(\theta_{FDP_{-}MCP}\right)+F_{FDS}\sin\left(\theta_{FDS_{-}MCP}\right)+F_{RI}\sin\left(\theta_{RI_{-}MCP}\right)\\ +F_{UI}\sin\left(\theta_{UI_{-}MCP}\right)+F_{LU}\sin\left(\theta_{LU_{-}MCP}\right)+F_{LE}\sin\left(\theta_{LE_{-}MCP}\right)\\ -F_{X_{-}MCP}=0\\ F_{FDP}\cos\left(\theta_{FDP_{-}MCP}\right)+F_{FDS}\cos\left(\theta_{FDS_{-}MCP}\right)+F_{RI}\cos\left(\theta_{RI_{-}MCP}\right)\\ +F_{UI}\cos\left(\theta_{UI_{-}MCP}\right)+F_{LU}\cos\left(\theta_{LU_{-}MCP}\right)+F_{LE}\cos\left(\theta_{LE_{-}MCP}\right)\\ +P_{Z}-F_{Z_{-}MCP}=0\\ F_{FDP}R_{FDP_{-}MCP}+F_{FDS}R_{FDS_{-}MCP}+F_{RI}R_{RI_{-}MCP}+F_{UI}R_{UI_{-}MCP}\\ +F_{LU}R_{LU_{-}MCP}-F_{LE}R_{LE_{-}MCP}=P_{Z}[L_{1}\sin\theta_{0}\\ +L_{2}\sin\left(\theta_{0}+\theta_{1}\right)+L_{3}\sin\left(\theta_{0}+\theta_{1}+\theta_{2}\right)] \end{array}\right.,$$

(2) $$PIP\left\{ \begin{array}{l} F_{FDP}\sin\left(\theta_{FDP_{-}PIP}\right)+F_{FDS}\sin\left(\theta_{FDS_{-}PIP}\right)+F_{ES}\sin\left(\theta_{ES_{-}PIP}\right)\\ +F_{UB}\sin\left(\theta_{UB_{-}PIP}\right)+F_{RB}\sin\left(\theta_{RB_{-}PIP}\right)-F_{X_{-}PIP}=0\\ F_{FDP}\cos\left(\theta_{FDP_{-}PIP}\right)+F_{FDS}\cos\left(\theta_{FDS_{-}PIP}\right)+F_{ES}\cos\left(\theta_{ES_{-}PIP}\right)\\ +F_{UB}\cos\left(\theta_{UB_{-}PIP}\right)+F_{RB}\cos\left(\theta_{RB_{-}PIP}\right)+P_{Z}-F_{Z_{-}PIP}=0\\ F_{FDP}R_{FDP_{-}PIP}+F_{FDS}R_{FDS_{-}PIP}-F_{ES}R_{ES_{-}PIP}-F_{UB}R_{UB_{-}PIP}-F_{RB}R_{RB_{-}PIP}\\ =P_{Z}\left[L_{1}\sin\theta_{0}+L_{2}\sin\left(\theta_{0}+\theta_{1}\right)\right] \end{array}\right.,$$

(3) $$DIP\{\begin{array}{l} F_{FDP}\sin\left(\theta_{FDP_{-}DIP}\right)+F_{IE}\sin\left(\theta_{TE_{-}DIP}\right)-F_{X_{-}DIP}=0\\ F_{FDP}\cos\left(\theta_{FDP_{-}DIP}\right)+F_{IE}\cos\left(\theta_{TE_{-}DIP}\right)+P_{Z}-F_{Z_{-}DIP}=0\\ F_{FDP}R_{FDP_{-}DIP}-F_{TE}R_{TE_{-}DIP}=P_{Z}L_{1}\sin\theta_{0} \end{array},$$

where $F_{i}$ represents the muscle strength of each joint, $\theta_{i}$ represents the angle between the joint and the Z direction, $R_{i}$ represents the moment arm at the joint, and $P_{Z}$ represents the contact force of the fingertips. 

To estimate muscle strength, the force arm needs to be solved. The force arm of $R_{LE_{-}MCP}$, $R_{ES_{-}PIP}$, and $R_{TE_{-}DIP}$ can be solved using the following formula:

(4) $$R=\pm \frac{dr\cdot \theta_{c}}{d\theta_{c}}=\pm r,$$

where r represents the rotation radius of a certain joint, and $\theta_{c}$ represents the joint rotation angle.

The force arm of $R_{FDP_{-}MCP}$ and $R_{FDS_{-}PIP}$ can be solved using the following formula:

(5) $$R=\pm \frac{d\left(\theta_{c}h+2y\left(1-\left(\theta_{c}/2\right)/\tan\left(\theta_{c}/2\right)\right)\right)}{d\theta_{c}},$$

where h represents the distance from the straight part of the muscle tendon to its long axis, and y represents the distance from the end of the tendon of the muscle to the center of the joint.

The force arm of $R_{RI_{-}MCP}$, $R_{UI_{-}MCP}$, and $R_{UB_{-}PIP}$ can be solved using the following formula:

(6) $$R=\pm \frac{d\left(r^{0}+r^{1}\theta_{c}\right)\theta_{c}}{d\theta_{c}},$$

where $r^{0}$ and $r^{1}$ represent joint radius coefficients.

The force arm of $R_{LU_{-}MCP}$ can be solved using the following formula:

(7) $$R=\pm \frac{d\left[\left(r^{0}+r^{1}\theta_{c}\right)\theta_{c}-E_{FDP\_MCP}\right]}{d\theta_{c}},$$

where $E_{FDP\_MCP}$ represents the displacement of the muscle joint.

There are 16 unknowns in the finger balance equation, but only nine sets of equations cannot be solved directly. The problem needs to be transformed into a constraint-based optimal solution. The constraint equation can be constructed according to the biomechanical relationship of fingers [32], as shown below.

(8) $$\{\begin{array}{l} F_{RB}=\frac{2}{3}F_{LU}+\frac{1}{6}F_{LE}\\ F_{UB}=\frac{1}{3}F_{UI}+\frac{1}{6}F_{LE}\\ F_{TE}=F_{RB}+F_{UB}\\ F_{ES}=\frac{1}{3}F_{RI}+\frac{1}{3}F_{UI}+\frac{1}{3}F_{LU}+\frac{1}{6}F_{LE}\\ F_{LE}=F_{ES} \end{array}.$$

The optimized objective function is based on the principle of the minimum sum of squares of muscle stress, which is expressed as follows:

(9) $$J=\min\sum_{i=1}^{k}{\left(F_{i}/PCSA_{i}\right)}^{2},$$

where $F_{i}$ is the muscle strength of the muscle group, *PCSA* is the physiological cross-sectional area of muscle, as shown in Table 1, and k is the number of muscle groups.

The Lagrange multiplier was constructed, and then the constraint optimization was carried out. Finally, the estimated relationship of fingertip pressure for each muscle group could be obtained, as shown in Table 2.

It can be seen from the table that different muscle groups have muscle strength several times the pressure of the fingertip. According to the constructed corresponding relationship between fingertip pressure and muscle group strength, the rehabilitation assessment can be carried out through the fingertip pressure during training.

#### 3.1.3. Finger Strength Analysis

**Figure 10:**
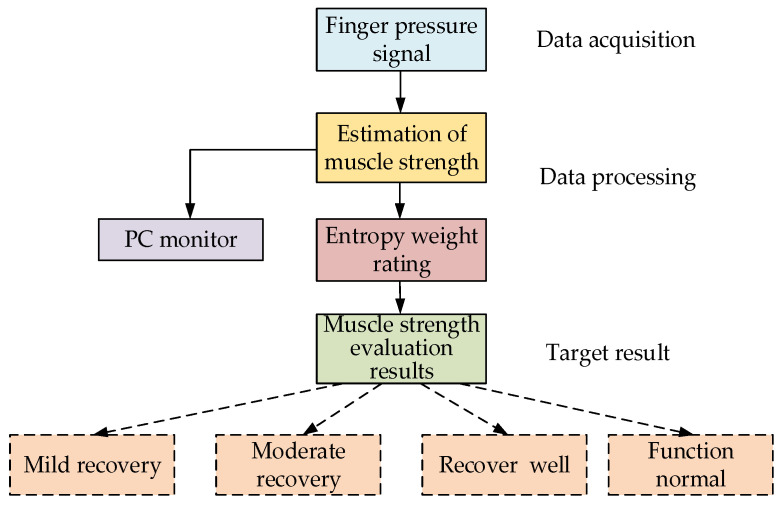
Muscle strength assessment model.

In this paper, the muscle strength was estimated from the collected fingertip pressure. After that, the estimated muscle strength was scored using the entropy weight method. The rehabilitation fingers were rated according to the score value [36]. The entire assessment model is shown in Figure 10.

The general steps of the entropy method are given below.

Firstly, the data need to be standardized. For data $X_{i}=\left\{x_{1},x_{2},x_{3},x_{4},\cdots ,x_{n}\right\}$, the standardization method can be expressed as

(10) $$Y_{ij}=\frac{X_{ij}-\min\left(X_{i}\right)}{\max\left(X_{i}\right)-\min\left(X_{i}\right)}.$$

Then, the proportion of each item can be determined as follows:

(11) $$P_{ij}=\frac{X_{ij}}{\sum_{i=1}^{n}X_{ij}}.$$

Next, the entropy values of each are calculated as follows:

(12) $$e_{j}=-k\times \sum_{i=1}^{n}P_{ij}\log\left(P_{ij}\right),$$

where $k=\frac{1}{\ln\left(n\right)}$.

After that, the coefficient of difference is expressed as follows:

(13) $$g_{j}{=1-e}_{j}.$$

Then, its weights are taken as follows:

(14) $$W_{j}=\frac{g_{j}}{\sum_{j=1}^{m}g_{j}}.$$

Finally, it can be scored as follows:

(15) $$S_{i}=\sum_{j=1}^{m}W_{j}\times P_{ij}.$$

**Figure 11:**
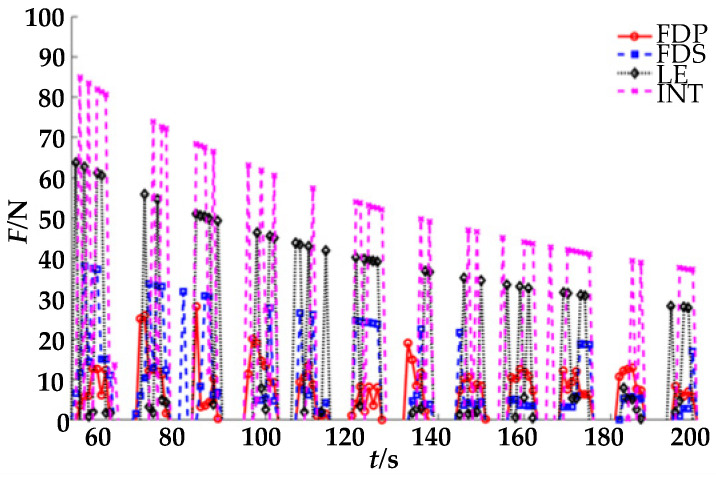
Four-fingertip pressure signals and muscle strength estimation results.

According to the above, flexion rehabilitation exercises were performed on the four fingers, and the fingertip pressure of the four fingers were collected. Then, the muscle strength was estimated on the basis of the four-finger pressure; the results are shown in Figure 11. In the rehabilitation process, although there were systematic errors caused by incomplete contact between the fingertip and finger-cot, physical displacement during movement, and finger-cot size, the entropy weight scoring method had a certain compensation ability, allowing a better estimation of the muscle strength of the FDP, FDS, LE, and INK muscle groups. Thus, the rehabilitation effect of people could be evaluated using the established estimation model of finger muscle strength.

**Figure 12:**
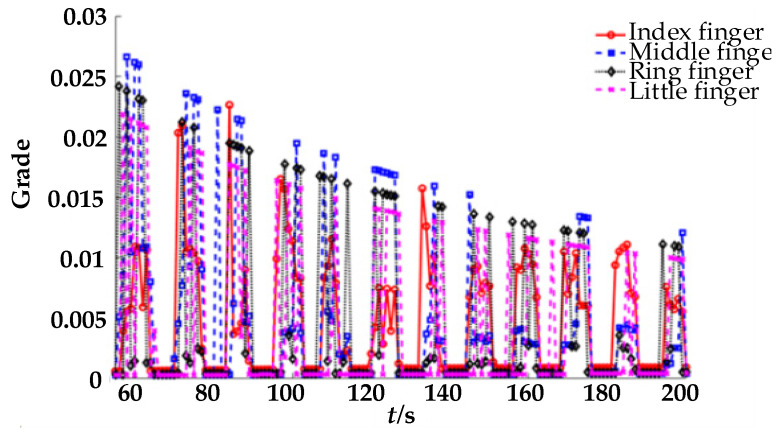
Muscle strength score of four fingers.

The estimated muscle strength of each finger was scored using the entropy method, and the results are shown in Figure 12. After grading the estimated muscle strength of each finger using the entropy weight method with the boundaries of 0.005, 0.010, and 0.015, the muscle strength level of each muscle group could be more intuitively understood.

### 3.2. Fatigue Assessment Based on sEMG Signal

#### 3.2.1. sEMG Signal Acquisition

**Figure 13:**
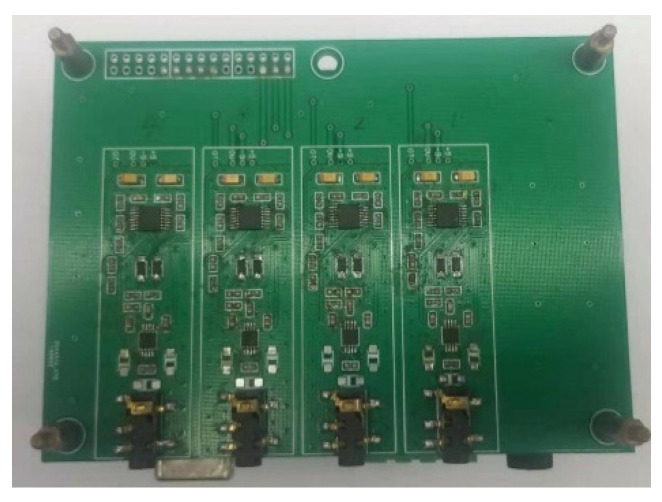
sEMG acquisition equipment.

**Figure 14:**
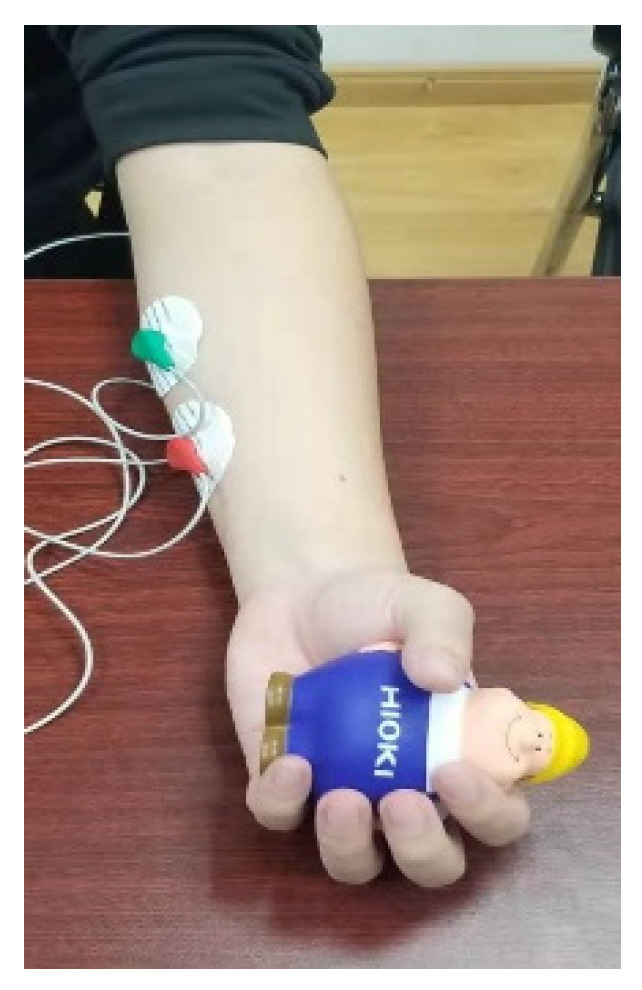
Muscle group location.

In finger rehabilitation training, muscle fatigue increases with training time, which not only does not guarantee the rehabilitation effect, but also affects the accuracy of rehabilitation assessment. This paper collected the sEMG signals of limb muscles to evaluate the fatigue degree of people during training to adjust the assessment process as a compensation factor. In this paper, the superficial flexor muscles of the forearm were selected [37], the sEMG acquisition equipment was developed as shown in Figure 13, and the muscle group location was as shown in Figure 14.

**Figure 15:**
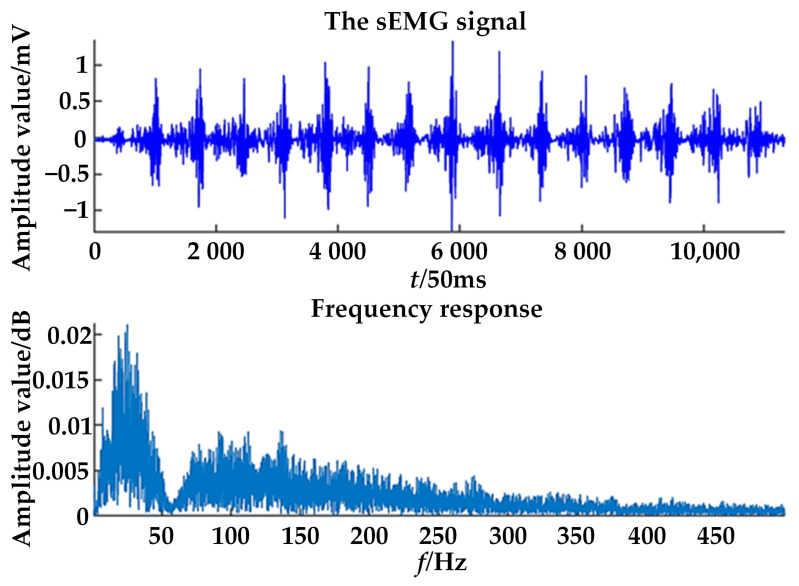
sEMG signals collected during flexion.

In this paper, the electrode used for sEMG signal acquisition was an electrocardiogram(ECG) patch. During the first flexion of the four fingers, the sEMG signal was collected once [38]. The sEMG signals collected are shown in Figure 15.

#### 3.2.2. Data Processing

The collected sEMG signal needed to be filtered first to improve the overall signal-to-noise ratio of sEMG. The filtering could be processed using second-order differential filtering, expressed as follows:

(16) $$x_{t}=y_{t+2}-y_{t+1}-y_{t}+y_{t-1},$$

where $x_{t}$ represents the filtered sEMG signal data, and $y_{t}$ represents the original sEMG signal data.

**Figure 16:**
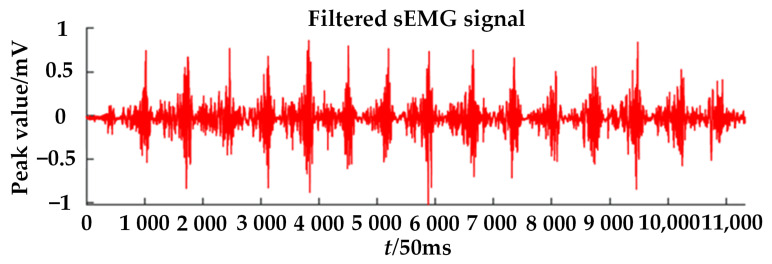
Filtered sEMG signal.

**Figure 17:**
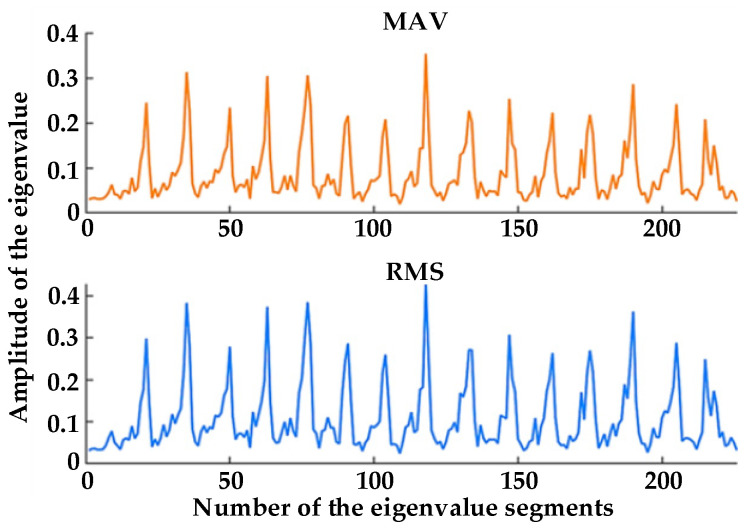
Feature extraction results.

The filtered sEMG signal is shown in Figure 16. In this paper, two eigenvalues of mean absolute value (MAV) and root mean square (RMS) were selected, the formulas of which are as follows [39]:

(17) $$\mathrm{MAV}=\frac{1}{N}\sum_{i=1}^{N}\left|x_{i}\right|,$$

(18) $$\mathrm{RMS}=\sqrt{\frac{1}{N}\sum_{i=1}^{N}x_{i}^{2}},$$

where N represents the data number of the sEMG signal in the time window, and $x_{i}$ represents the amplitude of the *i*-th sEMG signal. Here, N was selected as 50, and the eigenvalues of the extracted sEMG signal were as shown in Figure 17.

#### 3.2.3. Fatigue Degree Analysis

This paper selected 20 healthy volunteers (sex ratio 1:1, with ages of 20–50 and body mass index (BMI) of 19–23) to conduct an experiment to analyze the changes in eigenvalues. All volunteers gave their informed consent for inclusion before starting the experiment. First, the four-finger pressure and the sEMG signal data of the volunteers before training were collected, and then the volunteers carried out continuous active rehabilitation training for 20 min. After training, the finger pressure and sEMG data were collected again. Then, filtering processing and eigenvalue extraction were applied to the two sEMG signals.

**Figure 18:**
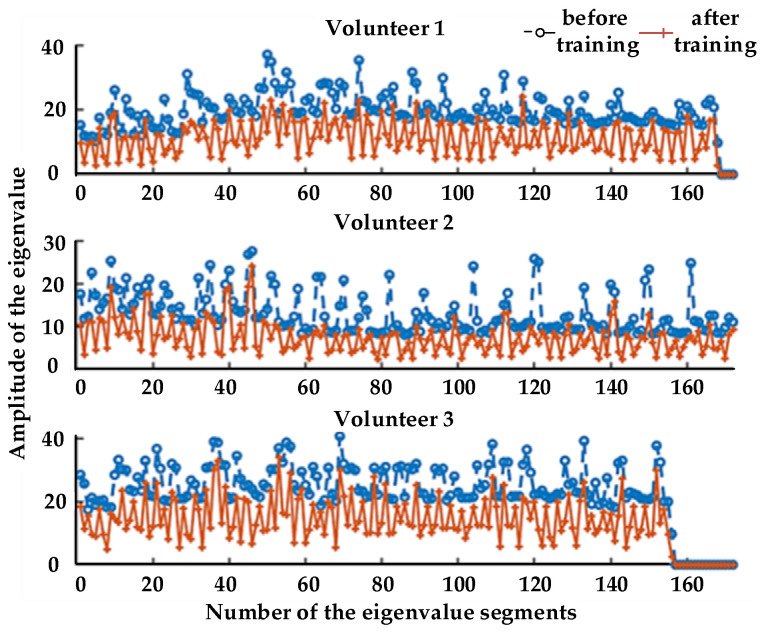
Changes in MAV.

**Figure 19:**
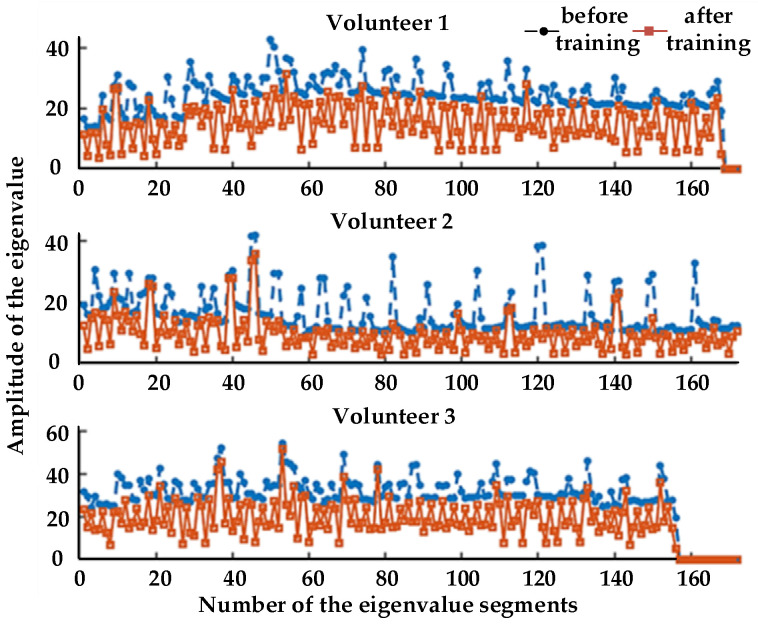
Changes in RMS.

The changes between the eigenvalues before and after training were analyzed. The extracted eigenvalues of three volunteers are shown in Figure 18 and Figure 19.

It can be seen that, during training, with the increase in fatigue degree, the eigenvalues of MAV and RMS of the three volunteers showed a certain degree of decline, which indicates that a certain relationship between fatigue degree and MAV and RMS. Therefore, we can judge the state of muscle fatigue evaluated on a four-point scale by analyzing their physiological signal data, corresponding to no fatigue, mild fatigue, moderate fatigue, and heavy fatigue.

### 3.3. Assessment of Range of Motion Based on Motor Encoder

#### 3.3.1. Human–Machine Motion Coupling Model

**Figure 20:**
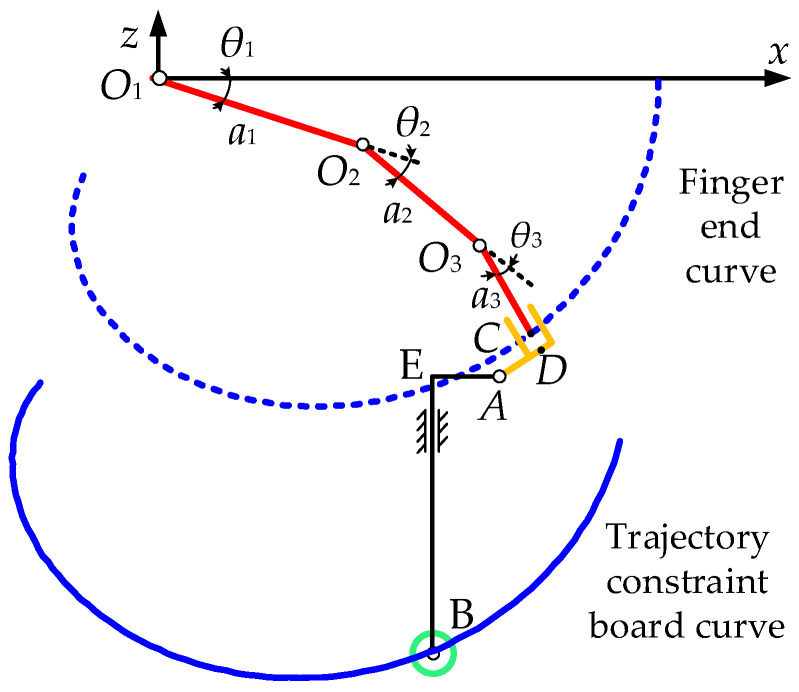
Motion mechanism diagram of four finger flexion/extension motion assembly.

The simplified model of the motion mechanism of the four-finger flexion/extension motion component is shown in Figure 20. The rolling bearing B moves in the groove of the finger track constraint plate, and its structure is similar to a moving cam. The natural movement track of the finger can be reproduced on the finger-cot D. Firstly, the finger-end trajectory can be obtained as a function of the size and motion coupling relationship of the finger, and then the finger motion trajectory constraint plate trajectory can be obtained by combining the size of the connecting rod EB.

It is stipulated that the joints of DIP, PIP, and MCP reach the maximum angle evenly and synchronously, i.e., the motion angles of the three joints have the following angle relationship [40]:

(19) $$\theta_{\mathrm{PIP}}=1.57\theta_{\mathrm{DIP}},$$

(20) $$\theta_{\mathrm{DIP}}=\theta_{\mathrm{MCP}},$$

where $\theta_{2}=1.57\theta_{3}$, and $\theta_{1}=\theta_{3}$.

In Figure 20, the length of CD is 10 mm, the length of AD is 15 mm, the length of AE is 11 mm, and the length of EB is 123 mm. The trajectory of the constraint board could be obtained by translating the trajectory of point A in the plane xoz along the axis x negative direction, i.e., the length of AE, and then along the axis z negative direction, i.e., the length of EB.

The trajectory parameter equation of point C at the end of the finger could be expressed as follows:

(21) $$\{\begin{array}{l} x_{C}=a_{3}\cos\left(3.57\theta_{3}\right)+a_{2}\cos\left(2.57\theta_{3}\right)+a_{1}\cos\theta_{3}\\ z_{C}=-\left[a_{3}\sin\left(3.57\theta_{3}\right)+a_{2}\sin\left(2.57\theta_{3}\right)+a_{1}\sin\theta_{3}\right] \end{array}.$$

Furthermore, the trajectory parameter equation of point B on the finger trajectory constraint board could be expressed as follows:

(22) $$\{\begin{array}{l} x_{B}=\left(a_{3}+10\right)\cos\left(3.57\theta_{3}\right)+a_{2}\cos\left(2.57\theta_{3}\right)+a_{1}\cos\theta_{3}-\left(a_{2}-10\right)\sin\left(3.57\theta_{3}\right)-11\\ z_{B}=-\left(a_{3}+10\right)\sin\left(3.57\theta_{3}\right)-a_{2}\sin\left(2.57\theta_{3}\right)-a_{1}\sin\theta_{3}-\left(a_{2}-10\right)\cos\left(3.57\theta_{3}\right)-123 \end{array}.$$

#### 3.3.2. ROM Analysis

Range of motion (ROM) is the primary indicator for evaluating patients with muscle and nerve injury [41]. The ROM was taken as $R_{1}$, $R_{2}$, $R_{3}$, corresponding to the flexion/extension of MCP ($\theta_{1}$), PIP ($\theta_{2}$), and DIP ($\theta_{3}$). The average activity of a healthy person was taken as ${\bar{R}}_{1}$, ${\bar{R}}_{2}$, ${\bar{R}}_{3}$. The assessment indicator was based on a four-point system, and the score sheet is shown in Table 3.

The angle change value was obtained through the motor encoder, and the moving distance of the rolling bearing B along the axis x could be calculated through conversion of the ball screw drive. Then, $\theta_{1}$, $\theta_{2}$, $\theta_{3}$ could be obtained using Equations (19), (20), and (22), respectively.

### 3.4. Comprehensive Assessment of Finger Rehabilitation

The comprehensive assessment model was based on the scores of ROM, muscle strength, and fatigue degree. After using the analytic hierarchy process (AHP) assessment method to get the weight comparison value, the maximum eigenvalue was then calculated using column method to determine the weight of the ROM and the three indicators. The normalized ROM weight judgement matrix and the comprehensive assessment weight judgement matrix are shown in Table 4 and Table 5 respectively.

According to Table 4 and Table 5, the total score of the comprehensive assessment of finger motion function could be calculated as follows:

(23) $$Z=0.45\times \left(0.55S_{R1}+0.28S_{R2}+0.17S_{R3}\right)+0.46S_{MS}+0.09S_{FD},$$

where Z is the total score of the comprehensive assessment, and $S_{Ri},S_{MS},S_{FD}$ are the scores of joint ROM, finger muscle strength, and finger fatigue degree, respectively.

According to Equation (23), a comprehensive assessment index that can reflect the rehabilitation level of people can be obtained from the score. The relationship between the comprehensive assessment index and the degree at which it is located is shown in Table 3, Table 4, Table 5 and Table 6. Finally, an appropriate rehabilitation program can be assigned according to the degree of finger rehabilitation.

**Figure 21:**
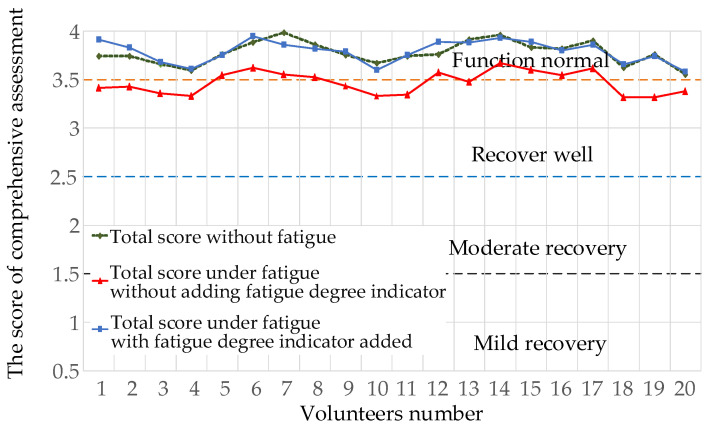
Comparison of comprehensive rehabilitation assessment.

The fatigue test data of 20 volunteers were introduced into the comprehensive assessment Equation (23) for correlation analysis, and the results are shown in Figure 21. The total score of the comprehensive assessment without fatigue showed that the degree of healthy volunteers was at the reasonable level of “normal function”; after a certain period of training, the comprehensive score decreased without adding the indicator of fatigue degree. After supplementing the calculation with the fatigue degree indicator, the total score after training tended to be consistent with the total score before training, and the error rate remained within 6.8%, proving that the assessment of comprehensive rehabilitation can be obtained from the training process and, thus, validating the feasibility of the assessment model.

## 4. Discussion

As shown in Figure 11, the pressure of the ring finger, middle finger, and little finger is high in the process of rehabilitation; the middle finger and ring finger are commonly used for force, whereas the contact between the pulp of the little finger and the finger-cot is not completely horizontal in the process of flexion. Therefore, there is a systematic error caused by the physical displacement of the little fingertip. At the same time, the reason for the small force of the index finger is that the finger-cot space is fixed and cannot fully fit each finger. Therefore, the gap between the index finger and the finger-cot is large, resulting in failure of the index finger to directly touch the pressure sensor throughout the rehabilitation process. According to the above problems, we can see that there were some defects in the finger-cot design of the rehabilitation robot, leading to certain errors in fingertip pressure detection during the rehabilitation process; this will be addressed in future generations of the robot for mechanism improvement.

Figure 18 and Figure 19 compare the eigenvalues of the three volunteers, where it can be seen that both MAV and RMS had a significant weakening trend before and after training. This shows that the effect was not caused by individual differences, whereas the embodiment of individual differences was mainly reflected in the size of their eigenvalues. The purpose of active training was to get the muscles to a state of fatigue, thereby highlighting the relationship between muscle fatigue and the MAV and RMS. Therefore, for the judgment of muscle fatigue, in addition to the subjective assessment of people, we can analyze their physiological signal data.

This paper designed a finger muscle strength estimation model based on fingertip pressure, a fatigue monitoring system based on sEMG signal, and a joint ROM estimation model based on a motor encoder, which provides a novel assessment method based on multi-sensor data. Compared with the existing common assessment methods, the comprehensive rehabilitation assessment method proposed in this paper can be completed automatically through multi-source data collection to help patients accurately locate their own rehabilitation situations, which has lower requirements for experienced rehabilitation doctors, and the collection method is simple and efficient. This has positive significance for realizing home treatment, thereby alleviating the shortage of rehabilitation doctors, reducing costs, and improving the efficiency of rehabilitation assessment.

## 5. Conclusions

In order to realize the objective, accurate, and convenient assessment of finger rehabilitation effect, a new finger rehabilitation assessment method was designed using the developed End-Effector Finger Rehabilitation Robot, which integrates muscle strength, fatigue degree, and joint ROM. Firstly, a finger muscle strength estimation model based on fingertip pressure was established, and the estimated muscle strength was scored and graded using the entropy weight method. Then, a fatigue monitoring system based on sEMG signal was designed to determine the fatigue degree of people in the training process by collecting the sEMG signals of muscles. Lastly, combined with the finger-joint ROM indicator, a comprehensive rehabilitation assessment model of finger function was established by using the AHP assessment method, which can achieve real-time assessment using multi-sensor signals in the process of rehabilitation training. The effectiveness of the rehabilitation assessment method was verified by experiment, providing the possibility of helping patients automatically, accurately locating the rehabilitation situations, and realizing convenient and objective finger rehabilitation assessment.

In the future, after optimizing the mechanical system and control system, the EFRR will be applied to real patient samples instead of healthy volunteers to further verify the feasibility of the comprehensive assessment method of finger function. Using the results of this comprehensive rehabilitation assessment method, an online system will be designed to realize home-based independent rehabilitation training and real-time rehabilitation assessment of patients.

## Figures and Tables

**Table 1:** Finger PCSA parameters (cm^2^).

Muscle Group	FDP	FDS	LE	LU	UI	RI
*PCSA*	10.67	8.89	4.15	0.52	1.45	4.30

**Table 2:** Muscle strength estimation results (N).

Muscle Group	FDP	FDS	LE	INT
Muscle strength	$2.5∼3P_{Z}$	$1.4∼2.8P_{Z}$	$0.7∼1.8P_{Z}$	$1.2∼3.7P_{Z}$

^1^ INT = RI + UI + LU.

**Table 3:** ROM rating scale of finger.

ROM	$0<R_{i}\leq 0.25{\bar{R}}_{i}$	$0.25<R_{i}\leq 0.5{\bar{R}}_{i}$	$0.5<R_{i}\leq 0.75{\bar{R}}_{i}$	$0.75<R_{i}\leq {\bar{R}}_{i}$
Score $S_{Ri}$	1	2	3	4

**Table 4:** Weight judgment matrix of ROM.

	$R_{1}$	$R_{2}$	$R_{3}$	Weight
$R_{1}$	1	1.93	3.15	0.55
$R_{2}$	0.52	1	1.66	0.28
$R_{3}$	0.32	0.6	1	0.17

**Table 5:** The comprehensive assessment weight judgement matrix.

	ROM	Muscle Strength	Fatigue Degree	Weight
ROM	1	1.44	0.23	0.45
Muscle strength	1.44	1	0.16	0.46
Fatigue degree	5	6.33	1	0.09

**Table 6:** Relationship between comprehensive assessment score and the degree of finger rehabilitation.

The Score of Comprehensive Assessment Z	0.5<Z≤1.5	1.5<Z≤2.5	2.5<Z≤3.5	3.5<Z≤4
Degree	Mild recovery	Moderate recovery	Good recovery	Normal function
